# In this month’s *Bulletin*

**DOI:** 10.2471/BLT.16.000916

**Published:** 2016-09-01

In the editorial section, Walter Johnson et al. (634) explain why a global response is needed to prevent and treat stroke. Bernadette Abela-Ridder et al. (635) announce a new rabies vaccine stockpile. 

Gary Humphreys and Fiona Fleck (638–639) report on efforts to address antimicrobial resistance, from national programmes to the United Nations General Assembly. Malcolm Molyneux tells Fiona Fleck (640–641) about the challenges countries face with the first introduction of a malaria vaccine. 

## El Salvador

## Preventing violent deaths

Charles M Katz et al. (660–666) model the interaction between imprisonment of gang members and homicide rates.

## Peru

## Screening eyesight

Sergio Latorre-Arteaga et al. (652–659) train teachers in rural communities to test children’s vision.

## South Sudan

## Tracking the toll of cholera

Cavin Epie Bekolo et al. (667–674) analyse vaccine use, disease severity and deaths during an outbreak.

## Global

## Treating malnourished children in hospital

Kirkby D Tickell and Donna M Denno (642–651) review WHO guidelines on severe acute malnutrition.

## Tracing an arbovirus

Mary Kay Kindhauser et al. (675–686) establish the historical record of Zika virus prior to the current epidemic.

## Paying for vaccines 

Tim Crocker-Buque and Sandra Mounier-Jack (687–693) gather stakeholders’ perspectives of a finance facility. 

## Improved finances, improved health 

Lois Orton et al. (694–704) review the evidence for health impacts of microfinance schemes.

## Detecting rare complications

Nirmal Kandel et al. (705–708) propose using polio surveillance systems for Zika-associated Guillain-Barré syndrome. 

